# Risk of stroke following SARS-CoV-2 infection in a nationwide self-controlled case series study in qatar

**DOI:** 10.1038/s41598-026-47216-z

**Published:** 2026-04-20

**Authors:** Hiam Chemaitelly, Naveed Akhtar, Salman Al Jerdi, Saadat Kamran, Sujatha Joseph, Deborah Morgan, Ryan Uy, Fatma Ben Abid, Abdullatif Al-Khal, Abdul-Badi Abou-Samra, Adeel A. Butt, Laith J. Abu-Raddad

**Affiliations:** 1 Infectious Disease Epidemiology Group, Weill Cornell Medicine-Qatar, Cornell University, Doha, Qatar; 2 Department of Population Health Sciences, Weill Cornell Medicine, Cornell University, New York, USA; 3 Neurosciences Institute, Hamad Medical Corporation, Doha, Qatar; 4 Department of Medical Education, Weill Cornell Medicine-Qatar, Cornell University, Doha, Qatar; 5 Infectious Disease Division, Hamad Medical Corporation, Doha, Qatar; 6 College of Medicine, QU Health, Qatar University, Doha, Qatar; 7 Corporate Quality and Patient Safety Department, Hamad Medical Corporation, Doha, Qatar; 8 Department of Medicine, Weill Cornell Medicine, Cornell University, New York, USA; 9 Department of Public Health, College of Health Sciences, QU Health, Qatar University, Doha, Qatar; 10 College of Health and Life Sciences, Hamad bin Khalifa University, Doha, Qatar

**Keywords:** Adverse event, COVID-19, Ischemic, Hemorrhagic, Risk, Thrombosis, Diseases, Health care, Medical research, Neurology, Risk factors

## Abstract

This study investigated stroke incidence after severe acute respiratory syndrome coronavirus 2 (SARS-CoV-2) infection using Qatar’s nationwide stroke registry linked with national testing, vaccination, and mortality databases. A self-controlled case series design was implemented among individuals with stroke and confirmed primary SARS-CoV-2 infection between January 1, 2020, and April 11, 2023. Incidence rate ratios (IRRs) comparing stroke incidence rate during the 1–90 days post-infection with predefined control periods were estimated using conditional Poisson regression. Of 4,187 stroke cases, 338 with confirmed infection met inclusion criteria. Stroke incidence rate was elevated during the 1–90 days post-infection (IRR 1.84, 95% CI: 1.31–2.58), peaking at days 1–28 (IRR 2.22, 95% CI: 1.53–3.22) and declining thereafter (days 29–59: IRR 1.47, 95% CI: 0.97–2.24; days 60–90: IRR 0.72, 95% CI: 0.43–1.21). This corresponded to 5.9 excess strokes per 100,000 infections. Elevated risk was confined to pre-Omicron infections (IRR 2.98, 95% CI: 1.89–4.70), with no evidence of increased risk observed for Omicron infections (IRR 0.82, 95% CI: 0.46–1.45). Subtype analyses showed increased risk for ischemic stroke (IRR 1.99, 95% CI: 1.37–2.89) and cerebral venous sinus thrombosis (CVST; IRR 3.52, 95% CI: 1.15–10.78), but not hemorrhagic stroke (IRR 0.64, 95% CI: 0.16–2.51). SARS-CoV-2 infection was associated with a transient elevation in stroke risk, peaking shortly after infection and declining thereafter. Excess risk was confined to pre-Omicron infections and driven primarily by ischemic stroke, while CVST, though rare, showed the strongest relative effect.

**Supplementary Information:**

The online version contains supplementary material available at 10.1038/s41598-026-47216-z.

## Introduction

Stroke is the second leading cause of death and the third leading cause of disability worldwide^1^. Reducing its burden is central to achieving the United Nations Sustainable Development Goal (SDG) of lowering premature mortality from non-communicable diseases by one-third by 2030^2^. Acute infections can trigger vascular events through systemic inflammation, endothelial dysfunction, and prothrombotic states, with elevated inflammatory markers linked to increased stroke risk^3^.

Since its emergence, severe acute respiratory syndrome coronavirus 2 (SARS-CoV-2) has continued to circulate globally, with recurrent surges driven by emerging variants and waning natural and vaccine immunity^4–6^. Evidence on the association between SARS-CoV-2 infection and subsequent stroke risk has been inconsistent, possibly reflecting differences in study design, population characteristics, and analytical methods^7–16^.

Several self-controlled case series (SCCS) studies have reported 2- to 7-fold increases in ischemic stroke risk within the first few weeks post-infection, including nationwide analyses from England^7,8^, the United States^9^, Sweden^10^, and Denmark^11^. Elevated risks of hemorrhagic stroke have also been observed shortly after infection^7,12^, along with markedly increased risks of cerebral venous sinus thrombosis (CVST)^8^ and venous thromboembolism^17^. In contrast, large retrospective cohort studies have found no increase in stroke risk^13,14^ or even lower risk^15,16^. Importantly, nearly all of these studies were conducted during the pre-Omicron phase of the pandemic.

Using national data from Qatar, this study investigated the incidence of stroke following primary SARS-CoV-2 infection using the SCCS method, a robust within-person design that inherently controls for time-invariant confounders^18,19^. Stroke incidence was evaluated during predefined post-infection risk windows relative to both pre- and post-infection control periods. Subgroup analyses assessed temporal patterns in risk, differences between pre-Omicron and post-Omicron periods, and variation by stroke subtype. Sensitivity analyses, including the use of different control windows, were conducted to assess the robustness of findings.

## Materials and methods

### Study population and data sources

This nationwide study was conducted in Qatar and included all individuals with a documented SARS-CoV-2-positive test result. Qatar’s SARS-CoV-2 testing program was extensive, with nearly 5% of the population tested weekly until November 1, 2022, after which testing rates declined to below 1% per week^20,21,22^. Most infections were identified through routine screening rather than symptom-driven testing^20,21^(Supplementary Section S1).

Stroke cases were identified from the Qatar Stroke Database, a prospective registry that has systematically recorded all admissions for acute ischemic or hemorrhagic stroke to Hamad Medical Corporation (HMC) since February 2013^23,24^ (Supplementary Section S2). HMC is the sole public tertiary healthcare provider in Qatar, operating a network of 15 hospitals and accounting for 98% of all acute stroke admissions nationwide^23^.

For this study, the stroke registry was linked to Qatar’s national federated databases on SARS-CoV-2 laboratory testing, vaccination, and all-cause mortality to retrieve individual-level histories of infection and vaccination, and to account for death as a censoring event (Supplementary Sections S1 and S3)^20,21^. Additional details on the study population and data sources have been reported previously^4,20,21,25–28,29^.

### Study design

A modified SCCS study^18,30^ was conducted to estimate the incidence of stroke following SARS-CoV-2 infection, using national data from Qatar spanning January 1, 2020, to April 11, 2023—the period of stroke data availability during the pandemic. This analytical framework inherently controls for all time-invariant confounders, as estimates are based solely on within-person variation in exposure status^18,19,30,31^.

**Fig. 1:**
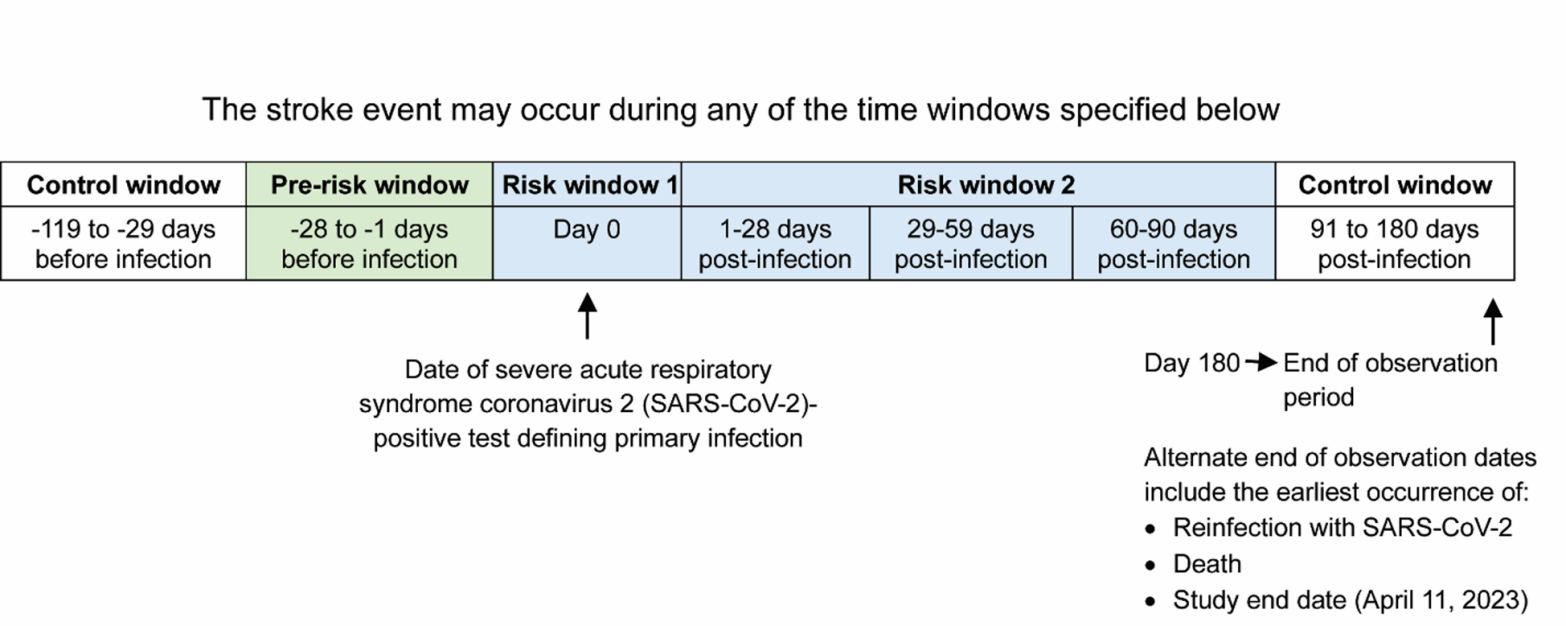
Design of the self-controlled case series study investigating the association between SARS-CoV-2 infection and stroke.

Exposure was defined strictly as a primary SARS-CoV-2 infection, operationalized as the first documented SARS-CoV-2-positive test since the onset of the pandemic. Reinfections were not considered exposures. The incidence rate (IR) of stroke was compared between two prespecified risk windows—day 0 and days 1–90 post-infection—and a control window comprising days 29–119 prior to infection and days 91–180 after infection (Fig. 1).

The definition of risk and control windows was guided by biological plausibility and supported by prior evidence^7–12^. Day 0 was treated as a distinct risk window to account for potential detection bias due to increased likelihood of SARS-CoV-2 testing during hospitalization for stroke^8,12,30^.

A pre-risk (buffer) window spanning the 1–28 days prior to infection was included in the analysis to account for the possibility that stroke may influence the likelihood of undergoing SARS-CoV-2 testing or acquiring infection (event-dependent exposure)^8,12,19^. Follow-up for each individual began 90 days before the pre-risk window and ended at the earliest occurrence of the following: SARS-CoV-2 reinfection, death, completion of the 180-day observation period following primary infection, or the study end date. Reinfection was defined as a SARS-CoV-2-positive test occurring ≥ 90 days after a prior infection, to avoid misclassifying prolonged viral shedding as reinfection^32,33,34^.

Only the first stroke event occurring during the study period was included in the analysis. To avoid misclassification of recurrent strokes as SARS-CoV-2-related incident events, individuals with a stroke diagnosis within the 365 days preceding their study stroke date were excluded^10,12,17,35^. Individuals without a recorded SARS-CoV-2-positive test during the study period and those whose stroke diagnosis fell outside the defined observation window were also excluded.

To reduce potential confounding from vaccine-associated stroke risk^17,35^, individuals who had received a COVID-19 vaccine within 49 days before stroke diagnosis were excluded^35^. This restriction was applied to support valid attribution of stroke events to SARS-CoV-2 infection. The 49-day window was specified a priori based on post-licensure pharmacovigilance investigations and SCCS-based safety studies, which identify a biologically plausible risk period for thrombotic and cerebrovascular events within the first 42 days following vaccination^35–39^, with rare reports extending to 45 days. Extending this interval by an additional week provided a conservative buffer to further reduce the likelihood that a vaccine-related stroke would be misattributed to SARS-CoV-2 infection.

### Statistical analysis

Descriptive characteristics of study participants were summarized using frequency distributions and measures of central tendency. Stroke IRs for each risk window and the control windows were calculated by dividing the number of stroke events occurring in the window by the total person-time (in days) contributed by all individuals to that window. IRs and their corresponding 95% confidence intervals (CIs) were estimated using a Poisson log-likelihood regression model, implemented via the *stptime* command in Stata version 18.0.

Incidence rate ratios (IRRs) and corresponding 95% CIs were estimated using a conditional Poisson regression model. IR of each risk window, as well as the pre-risk window, was compared to the control window.

To account for temporal variation in background stroke IR, calendar time was included as a time-varying covariate^19,31,35^. The midpoint of each risk, pre-risk, and control period was assigned to a calendar quarter of the year^35^. This adjustment controlled for potential time-varying confounding from seasonality, secular trends, and epidemic-related fluctuations^19,31,35^.

The SCCS method used in this study was adapted to account for event-dependent censoring due to the increased risk of death following stroke^18,30^. Specifically, the approach corrects for truncation of follow-up caused by post-stroke mortality by incorporating inverse-probability weights—estimated from the observed distribution of survival times after stroke—into the model as a log-transformed offset term^18,30^.

Subgroup analyses were conducted by stratifying the 1–90 day risk window into three intervals: days 1–28, 29–59, and 60–90 post-infection, to assess temporal variation in stroke risk following SARS-CoV-2 infection. Additional analyses estimated IRs and IRRs stratified by infection type (primary pre-Omicron infection vs. primary Omicron infection) and stroke type (ischemic, hemorrhagic, or CVST). Infections were classified as pre-Omicron if the SARS-CoV-2-positive test occurred before the onset of the Omicron wave in Qatar on December 19, 2021, and as Omicron otherwise^21^.

To further evaluate the robustness of the findings, two sensitivity analyses were performed. First, to address potential confounding by COVID-19 vaccination, individuals who received a vaccine dose between the date of SARS-CoV-2 infection and stroke diagnosis were excluded, irrespective of the interval between vaccination and stroke.

Second, because the primary analysis used both pre- and post-infection periods as the control window^8,10,12^, IRRs were re-estimated using either the pre-infection or the post-infection period alone to assess the impact of this methodological variation on the estimated outcomes.

The number of excess stroke cases attributable to SARS-CoV-2 infection was estimated using the SCCS model as^40,41^:

$$\:\mathrm{E}\mathrm{x}\mathrm{c}\mathrm{e}\mathrm{s}\mathrm{s}\:\mathrm{c}\mathrm{a}\mathrm{s}\mathrm{e}\mathrm{s}\:=\:\frac{IRR-1}{IRR}\times\:\mathrm{n}\mathrm{u}\mathrm{m}\mathrm{b}\mathrm{e}\mathrm{r}\:\mathrm{o}\mathrm{f}\:\mathrm{c}\mathrm{a}\mathrm{s}\mathrm{e}\mathrm{s}\:\mathrm{i}\mathrm{n}\:\mathrm{t}\mathrm{h}\mathrm{e}\:1-90\:\mathrm{d}\mathrm{a}\mathrm{y}\mathrm{s}\:\mathrm{p}\mathrm{o}\mathrm{s}\mathrm{t}-\mathrm{i}\mathrm{n}\mathrm{f}\mathrm{e}\mathrm{c}\mathrm{t}\mathrm{i}\mathrm{o}\mathrm{n}\:\mathrm{p}\mathrm{e}\mathrm{r}\mathrm{i}\mathrm{o}\mathrm{d}\:$$

This estimate reflects the number of excess stroke events attributable to SARS-CoV-2 infection among observed cases occurring within the prespecified risk window and should not be interpreted as necessarily a population-wide causal estimate. The excess cases were then standardized by dividing by the total number of primary infections during the study period and multiplying by 100,000, yielding the risk difference per 100,000 infections^40,41^.

CIs were not adjusted for multiplicity. Interactions were not investigated. Statistical analyses were conducted in STATA/SE version 18.0 (Stata Corporation, College Station, TX, USA, URL: www.https://www.stata.com).

### Ethics

The institutional review boards at Hamad Medical Corporation and Weill Cornell Medicine–Qatar approved this retrospective study with a waiver of informed consent. The study was conducted in accordance with the Declaration of Helsinki and reported following the Strengthening the Reporting of Observational Studies in Epidemiology (STROBE) guidelines (Table S1).

## Results

### Case population

**Fig. 2:**
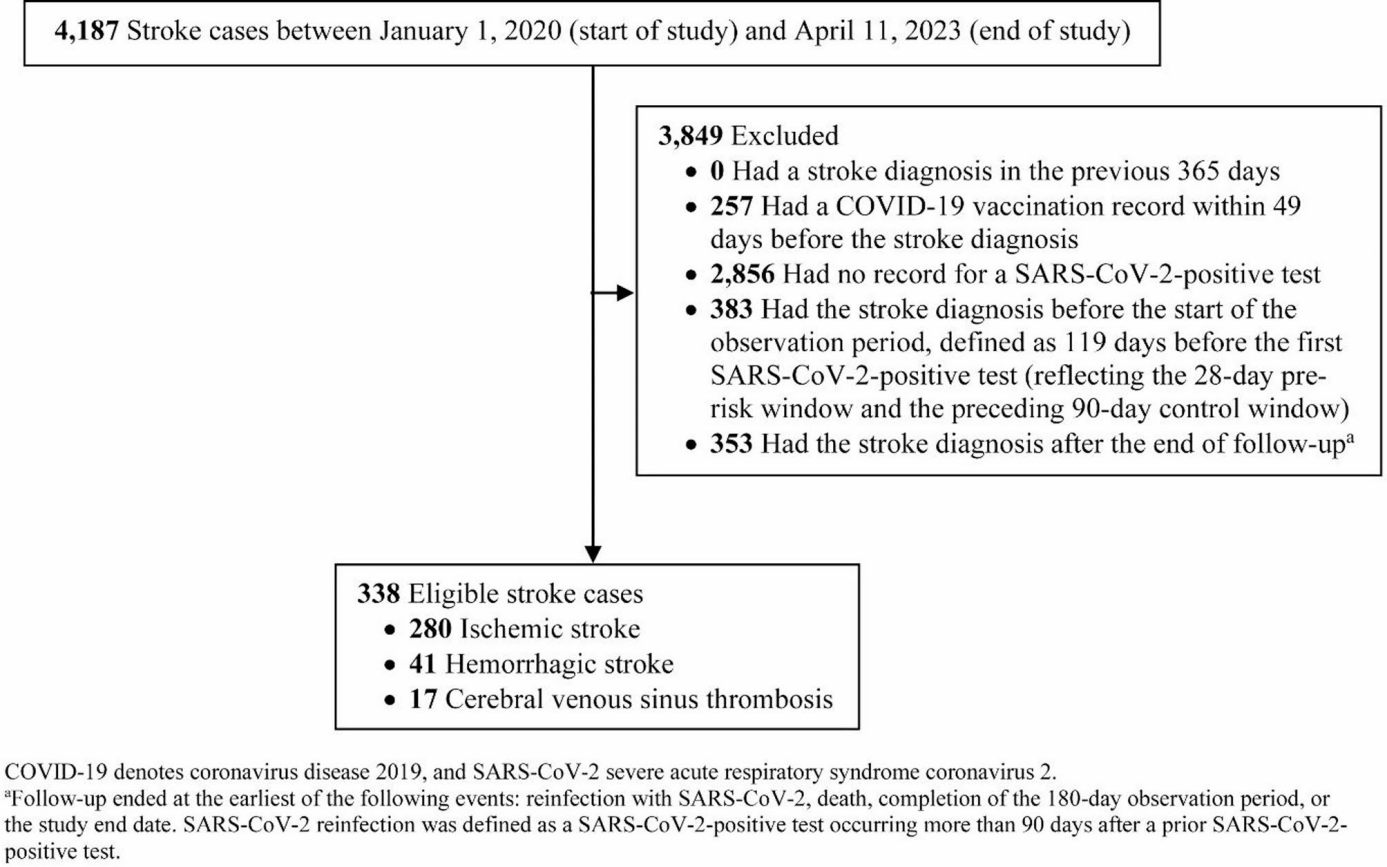
Selection of the study cohort for the self-controlled case series analysis of stroke incidence following SARS-CoV-2 infection.

The registry included 4,187 stroke cases during the study period (detailed description in Supplementary Section S4). Figure 2 illustrates the selection process for stroke cases included in the analysis of stroke incidence following SARS-CoV-2 infection. Table 1 summarizes the demographic, clinical, vaccination, case severity, and primary infection characteristics of the 338 eligible cases, including 280 ischemic strokes, 41 hemorrhagic strokes, and 17 CVST cases (detailed description in Supplementary Section S4).

**Table 1:** Characteristics of the cohort used to investigate stroke incidence following SARS-CoV-2 infection.

Characteristics	*N* = 338 (%)
Demographic characteristics	
Median age (IQR) — years	55 (45–65)
Age group — n (%)	
<40	41 (12.1)
40–49	79 (23.4)
50–59	90 (26.6)
60–69	69 (20.4)
≥70	52 (15.4)
Missing	7 (2.1)
Sex	
Male	239 (70.7)
Female	99 (29.3)
Nationality^a^	
Bangladeshi	45 (13.3)
Egyptian	10 (3.0)
Filipino	30 (8.9)
Indian	58 (17.2)
Nepalese	20 (5.9)
Pakistani	14 (4.1)
Qatari	78 (23.1)
Sri Lankan	6 (1.8)
Sudanese	13 (3.8)
Other nationalities^b^	64 (18.9)
Clinical characteristics	
Body mass index	
Underweight	4 (1.2)
Normal	117 (34.6)
Overweight	124 (36.7)
Obese	93 (27.5)
Smoking	
Non-smoker	271 (80.2)
Ex-smoker	53 (15.7)
Smoker	14 (4.1)
Diabetes mellitus	
No	107 (31.7)
Yes	176 (52.1)
Pre-diabetes	55 (16.3)
Hypertension	
No	117 (34.6)
Yes	221 (65.4)
Dyslipidemia	
No	232 (68.6)
Yes	106 (31.4)
Previous transient ischemic attack	
No	336 (99.4)
Yes	2 (0.6)
Previous deep vein thrombosis	
No	334 (98.8)
Yes	4 (1.2)
Atrial fibrillation	
No	316 (93.5)
Yes	22 (6.5)
Cardiac disease	
No	297 (87.9)
Yes	41 (12.1)
Renal failure	
No	315 (93.2)
Yes	23 (6.8)
Migraine	
No	337 (99.7)
Yes	1 (0.3)
Number of vaccine doses before the stroke event (> 49 days)	
0	214 (63.3)
1	2 (0.6)
2	91 (26.9)
3	30 (8.9)
4	1 (0.3)
Last vaccine type	
Unvaccinated	214 (63.3)
BNT162b2	85 (25.1)
mRNA-1273	38 (11.2)
ChAdOx1 nCoV-19	1 (0.3)
Primary infection (Exposure)	
Primary infection type	
Pre-Omicron	178 (52.7)
Omicron	160 (47.3)
Stroke occurrence	
Pre-infection control window	72 (21.3)
Pre-risk period	60 (17.7)
Day 0	32 (9.5)
1–90 days	119 (35.2)
Post-infection control window	55 (16.3)
Median time between primary infection and subsequent stroke (IQR) — days	50 (17–105)
Median time between primary infection and death^c^ (IQR) — days	32 (14–61)
Stroke severity indicators	
NIHSS (score)	
No deficit (0)	52 (15.4)
Minor stroke (1–4)	126 (37.3)
Moderate stroke (5–15)	100 (29.6)
Moderate-Severe stroke (16–20)	24 (7.1)
Severe stroke (21)	36 (10.6)
Intravenous thrombolysis administered	
Yes	27 (8.0)
No	311 (92.0)
Thrombectomy	
Yes	9 (2.7)
No	329 (97.3)
mRS at discharge (score)	
Poor functional outcome (0–2)	194 (57.4)
Good functional outcome (3–6)	143 (42.3)
Not applicable^d^	1 (0.3)
Median time between stroke and death^c^ (IQR) — days	18 (7–26)

COVID-19 denotes coronavirus disease, IQR, interquartile range, mRS, modified Rankin Scale, NIHSS, National Institutes of Health Stroke Scale, and SARS-CoV-2, severe acute respiratory syndrome coronavirus 2.

^a^Nationalities were chosen to represent the most populous groups in Qatar.

^b^These comprise 25 other nationalities in Qatar.

^c^Median time was calculated among the 15 individuals who experienced stroke after SARS-CoV-2 infection and died during the observation period.

^d^Study ended prior to patient discharge.

### Incidence of stroke following SARS-CoV-2 infection

#### Main analysis

Stroke IR was elevated during the 1–90 day post-infection window compared to the control period (IRR 1.84, 95% CI: 1.31–2.58; Table 2 and Fig. S1). Subgroup analysis by time since infection showed a declining trend, with IRR peaking at 2.22 (95% CI: 1.53–3.22) during days 1–28, decreasing to 1.47 (95% CI: 0.97–2.24) during days 29–59, and further to 0.72 (95% CI: 0.43–1.21) during days 60–90.

**Table 2:** Incidence of stroke following SARS-CoV-2 infection.

	Number of adverse events		Event rate per 1,000 person-days		Incidence rate ratio (95% CI)^a^
	At risk	Control	At risk	Control	
Any stroke type					
Pre-risk period	60	127	6.34 (4.92 to 8.17)	2.20 (1.85 to 2.62)	1.86 (1.32 to 2.61)
Day 0	32	127	94.67 (66.95 to 133.88)	2.20 (1.85 to 2.62)	29.31 (19.16 to 44.85)
1–90 days	119	127	4.13 (3.45 to 4.94)	2.20 (1.85 to 2.62)	1.84 (1.31 to 2.58)
Subgroup analysis^b^					
1–28 days	58	127	6.27 (4.85 to 8.12)	2.20 (1.85 to 2.62)	2.22 (1.53 to 3.22)
29–59 days	41	127	4.14 (3.05 to 5.63)	2.20 (1.85 to 2.62)	1.47 (0.97 to 2.24)
60–90 days	20	127	2.06 (1.33 to 3.20)	2.20 (1.85 to 2.62)	0.72 (0.43 to 1.21)
Pre-Omicron infection					
Pre-risk period	26	54	5.22 (3.55 to 7.66)	1.76 (1.35 to 2.30)	1.93 (1.14 to 3.28)
Day 0	20	54	112.36 (72.49 to 174.16)	1.76 (1.35 to 2.30)	38.08 (21.60 to 67.15)
1–90 days	78	54	5.15 (4.13 to 6.43)	1.76 (1.35 to 2.30)	2.98 (1.89 to 4.70)
Subgroup analysis^b^					
1–28 days	45	54	9.28 (6.93 to 12.43)	1.76 (1.35 to 2.30)	3.99 (2.49 to 6.40)
29–59 days	24	54	4.62 (3.10 to 6.89)	1.76 (1.35 to 2.30)	2.08 (1.19 to 3.62)
60–90 days	9	54	1.77 (0.92 to 3.39)	1.76 (1.35 to 2.30)	0.79 (0.37 to 1.69)
Omicron infection					
Pre-risk period	34	73	7.59 (5.42 to 10.62)	2.70 (2.14 to 3.39)	1.74 (1.12 to 2.71)
Day 0	12	73	75.00 (42.59 to 132.06)	2.70 (2.14 to 3.39)	17.83 (8.91 to 35.66)
1–90 days	41	73	3.00 (2.21 to 4.07)	2.70 (2.14 to 3.39)	0.82 (0.46 to 1.45)
Subgroup analysis^b^					
1–28 days	13	73	2.96 (1.72 to 5.09)	2.70 (2.14 to 3.39)	0.72 (0.35 to 1.47)
29–59 days	17	73	3.62 (2.25 to 5.82)	2.70 (2.14 to 3.39)	0.80 (0.41 to 1.57)
60–90 days	11	73	2.39 (1.33 to 4.32)	2.70 (2.14 to 3.39)	0.53 (0.25 to 1.11)
Ischemic stroke					
Pre-risk period	55	101	7.02 (5.39 to 9.14)	2.12 (1.75 to 2.58)	2.15 (1.49 to 3.09)
Day 0	25	101	89.29 (60.33 to 132.14)	2.12 (1.75 to 2.58)	29.76 (18.46 to 47.98)
1–90 days	99	101	4.15 (3.41 to 5.06)	2.12 (1.75 to 2.58)	1.99 (1.37 to 2.89)
Subgroup analysis^b^					
1–28 days	51	101	6.65 (5.05 to 8.75)	2.12 (1.75 to 2.58)	2.56 (1.71 to 3.84)
29–59 days	33	101	4.04 (2.87 to 5.68)	2.12 (1.75 to 2.58)	1.56 (0.98 to 2.47)
60–90 days	15	101	1.88 (1.13 to 3.11)	2.12 (1.75 to 2.58)	0.73 (0.41 to 1.32)
Hemorrhagic stroke					
Pre-risk period	4	21	3.48 (1.31 to 9.28)	2.96 (1.93 to 4.54)	0.61 (0.19 to 2.01)
Day 0	4	21	97.56 (36.62 to 259.94)	2.96 (1.93 to 4.54)	13.74 (3.93 to 48.07)
1–90 days	12	21	3.42 (1.94 to 6.02)	2.96 (1.93 to 4.54)	0.64 (0.16 to 2.51)
Subgroup analysis^b^					
1–28 days	2	21	1.82 (0.45 to 7.26)	2.96 (1.93 to 4.54)	0.24 (0.04 to 1.33)
29–59 days	7	21	5.79 (2.76 to 12.14)	2.96 (1.93 to 4.54)	0.73 (0.18 to 2.92)
60–90 days	3	21	2.50 (0.80 to 7.74)	2.96 (1.93 to 4.54)	0.31 (0.06 to 1.50)
Cerebral venous sinus thrombosis					
Pre-risk period	1	5	2.10 (0.30 to 14.91)	1.68 (0.70 to 4.05)	1.60 (0.19 to 13.82)^c^
Day 0	3	5	176.47 (56.92 to 547.16)	1.68 (0.70 to 4.05)	111.97 (26.46 to 473.86)^c^
1–90 days	8	5	5.41 (2.71 to 10.82)	1.68 (0.70 to 4.05)	3.52 (1.15 to 10.78)^c^
Subgroup analysis^b^					
1–28 days	5	5	10.50 (4.37 to 25.24)	1.68 (0.70 to 4.05)	6.71 (1.92 to 23.40)^c^
29–59 days	1	5	1.97 (0.28 to 14.00)	1.68 (0.70 to 4.05)	1.08 (0.13 to 9.21)^c^
60–90 days	2	5	4.03 (1.01 to 16.12)	1.68 (0.70 to 4.05)	1.82 (0.35 to 9.40)^c^

CI denotes confidence interval, and COVID-19, coronavirus disease.

^a^Incidence rate ratio estimated using the modified self-controlled case series method with a conditional Poisson regression model, adjusting for event-dependent censoring^18,30^ and calendar time.

^b^Incidence rate ratios estimated using a conditional Poisson regression model stratified by risk window.

^c^Incidence rate ratio estimated without adjustment for calendar time due to low case counts.

Stroke IRR was highest at 29.31 (95% CI: 19.16–44.85) on the day of the SARS-CoV-2-positive test (Day 0).

Among 921,066 primary SARS-CoV-2 infections recorded during the study period, an estimated 54.3 stroke cases were attributable to infection within the 1–90 day post-infection window. This corresponds to a risk difference of 5.90 (95% CI: 3.06–7.91) excess stroke cases per 100,000 infections.

### Analysis by infection type

Stratified analysis by infection type indicated that the increased risk of stroke was primarily driven by pre-Omicron infections (Table 2). During the 1–90 day post-infection period, the IRR for stroke following pre-Omicron infection was 2.98 (95% CI: 1.89–4.70). In contrast, no evidence of an increased risk was observed following Omicron infection (IRR: 0.82; 95% CI: 0.46–1.45).

Among individuals with pre-Omicron infection, subgroup analysis by time since infection showed a waning pattern similar to the main analysis: the IRR was 3.99 (95% CI: 2.49–6.40) during days 1–28, declined to 2.08 (95% CI: 1.19–3.62) during days 29–59, and further declined to 0.79 (95% CI: 0.37–1.69) during days 60–90 (Table 2).

### Analysis by stroke type

Stratified analysis by stroke type indicated an increased IR during the 1–90 day post-infection period for ischemic stroke (IRR: 1.99; 95% CI: 1.37–2.89) and CVST (IRR: 3.52; 95% CI: 1.15–10.78), while no evidence of an association was observed for hemorrhagic stroke (IRR: 0.64; 95% CI: 0.16–2.51) (Table 2).

Subgroup analysis by time since infection showed a declining trend in ischemic stroke IR, consistent with the main analysis (Table 2). Meanwhile, low case counts for hemorrhagic stroke and CVST limited the ability to assess temporal trends.

### Sensitivity analyses

Sensitivity analysis excluding all individuals who received COVID-19 vaccination between the infection and stroke dates—regardless of the interval—yielded IRR estimates consistent with the main results (Table S2).

The sensitivity analysis comparing stroke IR during the 1–90 day post-infection period to each individual control window showed that, when only the pre-infection control period was used, the estimated IR was comparable to that of the control period (IRR 1.28, 95% CI: 0.69–2.35) (Table S3). In contrast, when only the post-infection control period was used, the IR during the same risk window was substantially elevated (IRR 5.60; 95% CI: 2.30–13.62).

## Discussion

This nationwide, registry-based SCCS study found that SARS-CoV-2 infection was associated with a two-fold increase in stroke risk, peaking immediately after infection and declining over time. These findings are consistent with early-pandemic SCCS studies demonstrating a transient elevation in stroke risk following infection^7,8–10,11,12^ and reinforce evidence that acute infection can act as a short-term vascular trigger through systemic inflammation, cytokine-driven hypercoagulability, endothelial injury or dysfunction, and prothrombotic pathways^3,9,42,^^43,44^.

Importantly, an estimated 54 strokes were attributable to SARS-CoV-2 infection over the study period, corresponding to nearly six excess cases per 100,000 primary infections. In contrast, a recent analysis in the same population estimated fewer than 0.6 excess stroke cases per 100,000 vaccinations^45^, indicating that infection carries a ten-fold higher short-term risk for stroke than vaccination. This contrast underscores infection as a vascular hazard and highlights vaccination—particularly for older adults and those with vascular comorbidities—as a critical intervention to reduce preventable stroke burden during future infection waves.

Stroke risk was significantly elevated during the first month after a pre-Omicron infection and declined gradually over time, whereas no evidence for an increased risk was observed following Omicron infections. This divergence is unlikely to be explained by differences in testing practices—which remained largely stable throughout most of the study period^4^—but is consistent with evidence that Omicron infections are clinically milder than those caused by earlier variants^46–49^. Taken together, these findings suggest that infection severity is an important determinant of stroke risk and are consistent with a causal association for pre-Omicron infections.

Subtype-specific analyses indicated that the excess risk was driven largely by ischemic stroke, the most common subtype, and by CVST, which showed the strongest relative effect despite small case numbers. These findings mirror earlier SCCS studies reporting transient elevations in ischemic stroke risk, ranging from two-fold in the United States^9^ to seven-fold in Denmark^11^, with similar patterns observed in Sweden and England^7^^,8,10^.

Similarly, the CVST signal echoes findings from England, which documented a sharp increase within the first two weeks post-infection^8^. In contrast, no excess risk was detected for hemorrhagic stroke in this study, although the limited statistical precision restricts interpretation. This aligns with prior evidence showing no increase in hemorrhagic stroke risk in the first month post-infection, aside from a brief elevation in the first week^12^. These findings support SARS-CoV-2 as a trigger of both arterial and venous thrombotic events, with ischemic stroke contributing most to the absolute burden and CVST, though rare, representing the strongest relative effect.

This study has limitations. First, the markedly elevated stroke risk observed on Day 0 likely reflects, at least in part, detection bias, a phenomenon well documented in the literature^8,10,12,30^. This excess may reflect both infections that contributed to stroke and cases of reverse causality, whereby incidental infections were identified through routine testing of patients admitted with stroke from other underlying causes^30^. In this context, the excess risk on Day 0 may reflect stroke *with* SARS-CoV-2 infection rather than stroke *due to* the infection. To mitigate this bias, Day 0 was analyzed separately as an independent risk window^8,12,30^.

Second, sensitivity analyses highlighted the influence of comparator choice, as the effect size varied depending on whether the pre-infection or post-infection period was used as the control. Estimates were higher when only the post-infection period was used, but largely attenuated when the pre-infection period served as the comparator, likely reflecting different sources of bias in each window. During the pre-infection period, prodromal stroke symptoms or underlying comorbidities may have increased healthcare contact and SARS-CoV-2 testing, inflating the apparent stroke IR before infection.

Conversely, the post-infection period is vulnerable to depletion-of-susceptibles, or the healthy survivor effect, whereby individuals most susceptible to vascular complications (e.g., frail, high-risk patients) may have already experienced stroke shortly after infection, leaving a lower-risk survivor population^26^. These opposing biases support the study’s strategy of incorporating both pre-infection and post-infection control periods, rather than relying on only one, to achieve a more balanced and accurate assessment of the relationship between infection and stroke.

Third, the relatively small number of cases constrained statistical precision in several subgroup analyses, particularly for CVST and hemorrhagic stroke, leading to wide CIs. Fourth, exposure was defined as a primary SARS-CoV-2 infection based on documented SARS-CoV-2 testing; however, undocumented prior infections cannot be fully excluded despite Qatar’s extensive national testing strategy^20–22^. If present, such misclassification would attenuate the observed association—especially during the Omicron period—by biasing effect estimates toward the null, given that prior infection confer partial immunity or mitigate severity at reinfection^4,50,51^.

Fifth, data on venous thromboembolic events were not available, precluding assessment of broader thromboinflammatory outcomes. Finally, the findings may not be generalizable to more recent SARS-CoV-2 variants or to populations other than that of Qatar.

This study has strengths. First, it leveraged a nationwide, validated stroke registry with prospectively collected data, ensuring near-complete capture of stroke cases at the population level and minimizing recall and selection bias. Linkage with national SARS-CoV-2 testing records—collected during a period of high testing intensity—enhanced exposure ascertainment and was complemented by integration with national vaccination and mortality databases, enabling complete follow-up and accounting for the potential effect of vaccination.

Second, key design features were implemented to reduce bias. Day 0 was treated as a distinct risk window to minimize detection bias^30^, and a 28-day pre-risk buffer was incorporated to mitigate reverse causality, whereby prodromal manifestations of stroke could increase the likelihood of SARS-CoV-2 testing or nosocomial acquisition, leading to misclassification of exposure timing^19,30^.

Third, the SCCS design provided a robust methodological framework, inherently controlling for all fixed, time-invariant confounders—such as genetic susceptibility, chronic comorbidities, and healthcare access—making it particularly well suited for assessing acute adverse events in real-world populations^19^,^52,53,54^. The design was further strengthened by adjustment for event-dependent censoring^18,30^, and incorporation of calendar time adjustment to account for temporal variation in background stroke risk and secular trends^19,31,35^. Collectively, these features enhance internal validity and support a more reliable interpretation of the observed association between SARS-CoV-2 infection and stroke.

Finally, the validity of these findings is underscored by comparison with earlier work in the same population using a case-control design^16^. That study, which could not account for all fixed confounders, did not identify an increased risk of stroke after infection and even suggested a protective effect of infection against stroke^16^—highlighting the advantage of the SCCS approach in capturing a true association.

## Conclusion

SARS-CoV-2 infection was associated with a transient elevation in stroke risk, peaking immediately after infection and waning gradually thereafter. This excess risk was confined to pre-Omicron infections and driven mainly by ischemic stroke, while CVST, although rare, showed the strongest relative increase. Ongoing surveillance of cerebrovascular outcomes remains critical, particularly given the potential emergence of future variants with uncertain clinical severity.

## Supplementary Information

Below is the link to the electronic supplementary material.

Supplementary Material 1

## Data Availability

The dataset of this study is a property of the Qatar Ministry of Public Health that was provided to the researchers through a restricted-access agreement that prevents sharing the dataset with a third party or publicly. The data are available under restricted access for preservation of confidentiality of patient data. Access can be obtained through a direct application for data access to His Excellency the Minister of Public Health (https://https://emsfsa.moph.gov.qa/en/Pages/eservices.aspx). The raw data are protected and are not available due to data privacy laws. Requests for access are assessed by the Ministry of Public Health in Qatar, and approval is granted at its discretion. In compliance with data privacy laws and the data-sharing agreement with the Ministry of Public Health in Qatar, no datasets, whether raw or de-identified, can be publicly released by the researchers. Aggregate data are available within the paper and its supplementary information.
