# Supplementary material for: Risk of stroke following SARS-CoV-2 infection in a nationwide self-controlled case series study in qatar
Source: Sci Rep. 2026 Apr 20;16:18297. doi: 10.1038/s41598-026-47216-z (PMC13261118; doi:10.1038/s41598-026-47216-z)
Supplement: Supplementary file 1 — Supplementary Material 1 [file 41598_2026_47216_MOESM1_ESM.docx]

**Supplementary Information**

**Table of Contents**

[**Section S1. Study population and data sources** 2](#_Toc222246940)

[**Section S2. Qatar’s stroke registry** 5](#_Toc222246941)

[**Section S3. Laboratory methods and variant ascertainment** 6](#_Toc222246942)

[**Real-time reverse-transcription polymerase chain reaction testing** 6](#_Toc222246943)

[**Rapid antigen testing** 6](#_Toc222246944)

[**Section S4. Description of study Population** 8](#_Toc222246945)

[**Stroke cohort** 8](#_Toc222246946)

[**Case population** 8](#_Toc222246947)

[**Table S1.** **Strengthening the Reporting of Observational Studies in Epidemiology (STROBE) checklist for cohort studies.** 11](#_Toc222246948)

[**Fig. S1. Incidence rate of stroke following SARS-CoV-2 infection across study risk and control windows.** 13](#_Toc222246949)

[**Table S2. Sensitivity analysis. Incidence of stroke following SARS-CoV-2 infection, excluding individuals with COVID-19 vaccination between infection and stroke dates.^a^** 14](#_Toc222246950)

[**Table S3. Sensitivity analysis. Incidence of stroke following SARS-CoV-2 infection** **using either the pre-infection or post-infection period as the control window.** 15](#_Toc222246951)

[**References** 16](#_Toc222246952)

#

# **Section S1. Study population and data sources**

Qatar’s national and universal public healthcare system uses the Cerner Millenium electronic medical record (EMR) system to track all the public healthcare encounters of each individual in the country, including all citizens and residents registered in the national and universal public healthcare system. Registration in the public healthcare system is mandatory for citizens and residents.

The databases analyzed in this study are data-extract downloads from the national EMR database that have been implemented on a regular weekly schedule since the onset of pandemic by the Business Intelligence Unit at Hamad Medical Corporation (HMC). HMC is the national public healthcare provider in Qatar. At every download, all severe acute respiratory syndrome coronavirus 2 (SARS-CoV-2) tests, coronavirus disease (COVID-19) vaccinations, hospitalizations related to COVID-19, and all death records regardless of cause are provided to the authors through .csv files. These databases have been analyzed throughout the pandemic not only for study-related purposes, but also to provide policymakers with summary data and analytics to inform the national response.

Every health encounter in the national EMR is linked to an individual through the HMC Number, which serves as a unique identifier that links all records for this individual at the national level. Databases were merged and analyzed using the HMC Number to link all records pertaining to testing, vaccinations, hospitalizations, and deaths. All deaths in Qatar are recorded by the public healthcare system. COVID-19-related healthcare was provided exclusively in the public healthcare system. COVID-19 vaccination was also provided only through the public healthcare system. These health records were tracked throughout the COVID-19 pandemic using the national EMR system. This system has been implemented in 2013, before the onset of the pandemic. This pre-established system ensured that we had access to comprehensive health records related to this study for both citizens and residents throughout the entire pandemic, allowing us to follow each person over time.

Demographic details for every HMC Number (individual) such as sex, age, and nationality are collected upon issuing of the universal health card, based on the Qatar Identity Card, which is a mandatory requirement by the Ministry of Interior to every citizen and resident in the country. Data extraction from the Qatar Identity Card to the digital health platform is performed electronically through scanning techniques.

SARS-CoV-2 testing in any facility in Qatar is tracked nationally in one database, the national testing database. This database covers all testing throughout the country, whether in public or private facilities. Every polymerase chain reaction (PCR) test conducted in Qatar, regardless of location or setting, is classified on the basis of symptoms and the reason for testing, such as the presence of clinical symptoms, contact tracing, participation in surveys or random testing campaigns, individual requests for testing, routine healthcare testing, pre-travel requirements, at the point of entry into the country, or any other relevant reasons for testing.

Before November 1, 2022, SARS-CoV-2 testing in Qatar was performed extensively with about 5% of the population tested every week^1^. Based on the distribution of the reason for testing up to November 1, 2022, most of the tests in Qatar were conducted for routine reasons, such as travel-related purposes, and about 75% of infections were diagnosed not because of presence of symptoms^1,2^. Starting from November 1, 2022, testing for SARS-CoV-2 was substantially reduced with <1% of the population tested every week^2^. This study factored all SARS-CoV-2-related testing included in the national testing database over the duration of the study.

December 19, 2021 marked the onset of the Omicron wave in Qatar^1^. The first Omicron wave that reached its peak in January of 2022 was massive and strained the testing capacity in the country^1,3-5^. To alleviate the burden on PCR testing, rapid antigen testing was rapidly introduced. The swift change in testing policy precluded incorporating reason for testing for a number of rapid antigen tests. While the reason for testing is documented for all PCR tests, it is not uniformly available for all rapid antigen tests. However, all medically supervised rapid antigen tests were captured by the national integrated digital health platform since January 5, 2022.

Rapid antigen test kits are accessible for purchase at pharmacies in Qatar, but results of home-based testing are neither reported nor documented in the national databases. Since SARS-CoV-2-test outcomes were linked to specific public health measures, restrictions, and privileges, testing policy and guidelines stress facility-based testing as the core testing mechanism in the population. While facility-based testing is provided free of charge or at low subsidized costs, depending on the reason for testing, home-based rapid antigen testing is de-emphasized and not supported as part of national policy.

Qatar launched its COVID-19 vaccination program in December 2020, employing mRNA vaccines and prioritizing individuals based on coexisting conditions and age criteria^2,6^. COVID-19 vaccination was provided free of charge, regardless of citizenship or residency status, with early prioritization of older adults and individuals with comorbidities, and was nationally tracked^2,6^.

Qatar has unusually young, diverse demographics, in that only 9% of its residents are ≥50 years of age, and 89% are expatriates from over 150 countries^2,7^. Further descriptions of the study population and these national databases were reported previously^1,2,5,8-11^.

# **Section S2. Qatar’s stroke registry**

The registry contains detailed demographic and clinical information for each case, encompassing risk factors, laboratory and radiological findings, clinical presentation, and hospitalization course^12-14^. Stroke severity at presentation is assessed using the National Institutes of Health Stroke Scale (NIHSS), while stroke subtypes are classified according to the Trial of Org 10172 in Acute Stroke Treatment (TOAST) criteria^15^ and Bamford classification^16^. Additional variables captured include hospital length of stay and modified Rankin Scale (mRS) scores at pre-admission, discharge, and 90-day follow-up. All stroke diagnoses and outcomes are independently validated by licensed neurologists or stroke specialists.

# **Section S3. Laboratory methods and variant ascertainment**

## **Real-time reverse-transcription polymerase chain reaction testing**

Nasopharyngeal and/or oropharyngeal swabs were collected for PCR testing and placed in Universal Transport Medium (UTM). Aliquots of UTM were: 1) extracted on KingFisher Flex (Thermo Fisher Scientific, USA), MGISP-960 (MGI, China), or ExiPrep 96 Lite (Bioneer, South Korea) followed by testing with real-time reverse-transcription PCR (RT-qPCR) using TaqPath COVID-19 Combo Kits (Thermo Fisher Scientific, USA) on an ABI 7500 FAST (Thermo Fisher Scientific, USA); 2) tested directly on the Cepheid GeneXpert system using the Xpert Xpress SARS-CoV-2 (Cepheid, USA); or 3) loaded directly into a Roche cobas 6800 system and assayed with the cobas SARS-CoV-2 Test (Roche, Switzerland). The first assay targets the viral S, N, and ORF1ab gene regions. The second targets the viral N and E-gene regions, and the third targets the ORF1ab and E-gene regions.

All PCR testing was conducted at the HMC Central Laboratory or Sidra Medicine Laboratory, following standardized protocols.

## **Rapid antigen testing**

SARS-CoV-2 antigen tests were performed on nasopharyngeal swabs using one of the following lateral flow antigen tests: Panbio COVID-19 Ag Rapid Test Device (Abbott, USA); SARS-CoV-2 Rapid Antigen Test (Roche, Switzerland); Standard Q COVID-19 Antigen Test (SD Biosensor, Korea); or CareStart COVID-19 Antigen Test (Access Bio, USA). All antigen tests were performed at point-of-care according to each manufacturer's instructions, at public or private hospitals and clinics throughout Qatar, with prior authorization and training by the Ministry of Public Health (MOPH). Antigen test results were electronically reported to the MOPH in real time using the Antigen Test Management System which is integrated with the national COVID-19 database.

# **Section S4. Description of study Population**

## **Stroke cohort**

The registry included 4,187 stroke cases during the study period, of which 1,083 (25.9%) had a documented primary SARS-CoV-2 infection. Of these, 496 (45.8%) had SARS-CoV-2 infection before stroke diagnosis, 34 (3.1%) on the same day (Day 0), and 553 (51.1%) after the diagnosis. The median date of stroke diagnosis was September 14, 2021 (interquartile range [IQR]: October 24, 2020–March 3, 2022).

The median stroke diagnosis date was August 15, 2021 (IQR: October 29, 2020–June 25, 2022). Among individuals whose stroke occurred on the day of SARS-CoV-2 infection or afterwards, the median date of infection was March 20, 2021 (IQR: July 5, 2020–January 2, 2022), with a median interval of 282 days (IQR: 71–534) between infection and stroke diagnosis.

## **Case population**

Fig. 2 illustrates the selection process for stroke cases included in the analysis of stroke incidence following SARS-CoV-2 infection. Table 1 summarizes the demographic, clinical, vaccination, and primary infection characteristics of the 338 eligible cases, including 280 ischemic strokes, 41 hemorrhagic strokes, and 17 CVST cases.

The median age of the study population (eligible stroke cases) was 55.0 years (IQR: 45.0–65.0), and 70.7% were male. The most common nationalities were Qatari (23.1%), Indian (17.2%), and Bangladeshi (13.3%). Overweight and obesity were documented in 36.7% and 27.5% of cases, respectively. Current smoking was reported in 4.1% of cases, while 15.7% were ex-smokers. Comorbidities were prevalent: 52.1% had diabetes mellitus and 16.3% were pre-diabetic, 65.4% had hypertension, and 31.4% had dyslipidemia. Atrial fibrillation, cardiac disease, and renal failure were reported in 6.5%, 12.1%, and 6.8% of cases, respectively.

A total of 124 individuals (36.7%) had received a COVID-19 vaccine more than 49 days before their stroke event. Among these, 73.4% had completed the primary vaccination series, and 25.0% had received booster doses. The most recent vaccine received was BNT162b2 in 68.5% of cases, mRNA-1273 in 30.6%, and ChAdOx1 nCoV-19 in fewer than 1%.

Overall, 82.3% of strokes were classified as minor or moderate, while 17.7% were categorized as moderate–severe or severe. Acute reperfusion therapy was administered in a minority of cases, with 8.0% receiving intravenous thrombolysis and 2.7% undergoing mechanical thrombectomy. Functional status at discharge, assessed using mRS, indicated that 57.4% of patients experienced residual disability.

Primary SARS-CoV-2 infection was attributed to a pre-Omicron variant in 52.7% of cases and to an Omicron variant in 47.3%. Stroke occurred after infection in 51.5% of cases, on the same day in 9.5%, and before infection in 39.0%. Among individuals with post-infection stroke, the median date of primary infection was April 21, 2021 (IQR: September 8, 2020–January 10, 2022), with a median interval of 50 days (IQR: 17–105) between infection and stroke diagnosis.

A total of 24 stroke cases died during the observation period, including 15 individuals whose stroke occurred after SARS-CoV-2 infection, resulting in a case fatality rate of 7.3% in this subgroup. Among these 15 deaths, none occurred on Day 0, 7 occurred within 1–28 days post-infection, 4 between 29–59 days, 3 between 60–90 days, and 1 during the post-infection control window (91–180 days). Among these 15 individuals, the median time from stroke to death was 18 days (IQR: 7–26), and the median time from infection to death was 32 days (IQR: 14–61).

Four individuals experienced SARS-CoV-2 reinfection and were censored at the time of reinfection, 17 were censored at the end of the study period, and 293 completed the full 180-day follow-up.

# **Table S1.** **Strengthening the Reporting of Observational Studies in Epidemiology (STROBE) checklist for cohort studies.**

|  | Item No | Recommendation | Main Text page |
| --- | --- | --- | --- |
| **Title and abstract** | 1 | (*a*) Indicate the study’s design with a commonly used term in the title or the abstract | Title & Abstract |
|  |  | (*b*) Provide in the abstract an informative and balanced summary of what was done and what was found | Abstract |
| Introduction | | | |
| Background/rationale | 2 | Explain the scientific background and rationale for the investigation being reported | Background |
| Objectives | 3 | State specific objectives, including any prespecified hypotheses | Background |
| Methods | | | |
| Study design | 4 | Present key elements of study design early in the paper | Materials and Methods (‘Study design’) & Fig. 1 |
| Setting | 5 | Describe the setting, locations, and relevant dates, including periods of recruitment, exposure, follow-up, and data collection | Materials and Methods (‘Study population and data sources’ & ‘Study design’), Figs. 1-2, & Sections S1-S3 in Supplementary Information |
| Participants | 6 | (*a*) Give the eligibility criteria, and the sources and methods of selection of participants. Describe methods of follow-up | Materials and Methods (‘Study population and data sources’ & ‘Study design’) & Fig. 2 |
|  |  | (*b*) For matched studies, give matching criteria and number of exposed and unexposed |  |
| Variables | 7 | Clearly define all outcomes, exposures, predictors, potential confounders, and effect modifiers. Give diagnostic criteria, if applicable | Materials and Methods (‘Study design’ & ‘Statistical analysis’), Table 1, & Sections S1 & S3 in Supplementary Information |
| Data sources/ measurement | 8* | For each variable of interest, give sources of data and details of methods of assessment (measurement). Describe comparability of assessment methods if there is more than one group | Materials and Methods (‘Study population and data sources’, ‘Study design’, & ‘Statistical analysis’), Fig. 1, Table 1, & Sections S1-S3 in Supplementary Information |
| Bias | 9 | Describe any efforts to address potential sources of bias | Materials and Methods (‘Study design’ & ‘Statistical analysis’) |
| Study size | 10 | Explain how the study size was arrived at | Fig. 2 |
| Quantitative variables | 11 | Explain how quantitative variables were handled in the analyses. If applicable, describe which groupings were chosen and why | Materials and Methods (‘Statistical analysis’) & Table 1 |
| Statistical methods | 12 | (*a*) Describe all statistical methods, including those used to control for confounding | Materials and Methods (‘Statistical analysis’) |
|  |  | (*b*) Describe any methods used to examine subgroups and interactions | Materials and Methods (‘Statistical analysis’) |
|  |  | (*c*) Explain how missing data were addressed | Not applicable, see Materials and (‘Study population and data sources’) & Section S1 in Supplementary Information |
|  |  | (*d*) If applicable, explain how loss to follow-up was addressed | Not applicable, see Materials and (‘Study population and data sources’) & Section S1 in Supplementary Information |
|  |  | (*e*) Describe any sensitivity analyses | Materials and Methods (‘Statistical analysis’) |
| Results | | |  |
| Participants | 13* | (a) Report numbers of individuals at each stage of study—eg numbers potentially eligible, examined for eligibility, confirmed eligible, included in the study, completing follow-up, and analysed | Fig. 2 |
|  |  | (b) Give reasons for non-participation at each stage |  |
|  |  | (c) Consider use of a flow diagram |  |
| Descriptive data | 14 | (a) Give characteristics of study participants (eg demographic, clinical, social) and information on exposures and potential confounders | Results (‘Case population’), Table 1, and Section S4 in Supplementary Information |
|  |  | (b) Indicate number of participants with missing data for each variable of interest | Not applicable, see Materials and Methods (‘Study population and data sources’) & Section S1 in Supplementary Information |
|  |  | (c) Summarise follow-up time (eg, average and total amount) | Results (‘Case population’), Table 1, & Section S4 in Supplementary Information |
| Outcome data | 15 | Report numbers of outcome events or summary measures over time | Results (‘Incidence of stroke following vaccination -Main analysis’), Table 2 and Fig. S1 |
| Main results | 16 | (a) Give unadjusted estimates and, if applicable, confounder-adjusted estimates and their precision (eg, 95% confidence interval). Make clear which confounders were adjusted for and why they were included | Results (‘Incidence of stroke following vaccination -Main analysis’) and Table 2 |
|  |  | (b) Report category boundaries when continuous variables were categorized | Table 1 |
|  |  | (c) If relevant, consider translating estimates of relative risk into absolute risk for a meaningful time period | Results (‘Incidence of stroke following vaccination -Main analysis’) |
| Other analyses | 17 | Report other analyses done—eg analyses of subgroups and interactions, and sensitivity analyses | Results (‘Incidence of stroke following vaccination -Analysis by infection type & Analysis by stroke type’ & ‘Sensitivity analyses’) and Tables S2-S3 in Supplementary Information |
| Discussion | | | |
| Key results | 18 | Summarise key results with reference to study objectives | Discussion, paragraphs 1-5 |
| Limitations | 19 | Discuss limitations of the study, taking into account sources of potential bias or imprecision. Discuss both direction and magnitude of any potential bias | Discussion, paragraphs 6-10 |
| Interpretation | 20 | Give a cautious overall interpretation of results considering objectives, limitations, multiplicity of analyses, results from similar studies, and other relevant evidence | Conclusion |
| Generalisability | 21 | Discuss the generalisability (external validity) of the study results | Discussion, paragraph 10 |
| Other information | | | |
| Funding | 22 | Give the source of funding and the role of the funders for the present study and, if applicable, for the original study on which the present article is based | Funding |

# **Fig. S1. Incidence rate of stroke following SARS-CoV-2 infection across study risk and control windows.**


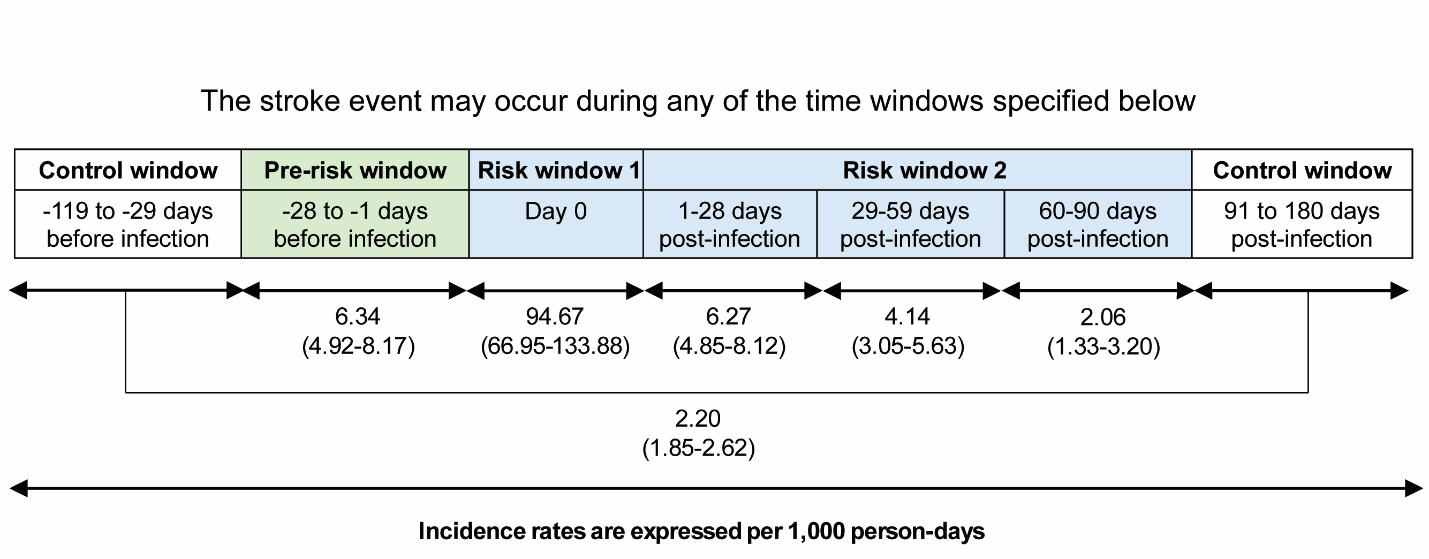


# **Table S2. Sensitivity analysis. Incidence of stroke following SARS-CoV-2 infection, excluding individuals with COVID-19 vaccination between infection and stroke dates.^a^**

|  | **Number of adverse events** | | **Event rate per 1,000 person-days** | | **Incidence rate ratio**  **(95% CI)**^b^ |
| --- | --- | --- | --- | --- | --- |
|  | **At risk** | **Control** | **At risk** | **Control** |  |
| **Any stroke type** |  |  |  |  |  |
| Pre-risk period | 60 | 124 | 6.40 (4.97 to 8.24) | 2.17 (1.82 to 2.59) | 1.89 (1.35 to 2.66) |
| Day 0 | 32 | 124 | 95.52 (67.55 to 135.08) | 2.17 (1.82 to 2.59) | 30.04 (19.60 to 46.04) |
| 1-90 days | 119 | 124 | 4.17 (3.48 to 4.99) | 2.17 (1.82 to 2.59) | 1.90 (1.35 to 2.68) |
| **Pre-Omicron infection** |  |  |  |  |  |
| Pre-risk period | 26 | 52 | 5.28 (3.59 to 7.75) | 1.72 (1.31 to 2.26) | 1.97 (1.16 to 3.35) |
| Day 0 | 20 | 52 | 113.64 (73.31 to 176.14) | 1.72 (1.31 to 2.26) | 38.90 (22.01 to 68.76) |
| 1-90 days | 78 | 52 | 5.21 (4.18 to 6.51) | 1.72 (1.31 to 2.26) | 3.06 (1.93 to 4.86) |
| **Omicron infection** |  |  |  |  |  |
| Pre-risk period | 34 | 72 | 7.64 (5.46 to 10.69) | 2.68 (2.13 to 3.37) | 1.77 (1.14 to 2.76) |
| Day 0 | 12 | 72 | 75.47 (42.86 to 132.89) | 2.68 (2.13 to 3.37) | 18.42 (9.20 to 36.90) |
| 1-90 days | 41 | 72 | 3.01 (2.22 to 4.09) | 2.68 (2.13 to 3.37) | 0.85 (0.48 to 1.52) |
| **Ischemic stroke** |  |  |  |  |  |
| Pre-risk period | 55 | 99 | 7.07 (5.42 to 9.20) | 2.10 (1.72 to 2.55) | 2.20 (1.53 to 3.17) |
| Day 0 | 25 | 99 | 89.93 (60.77 to 133.09) | 2.10 (1.72 to 2.55) | 30.65 (18.97 to 49.51) |
| 1-90 days | 99 | 99 | 4.18 (3.44 to 5.10) | 2.10 (1.72 to 2.55) | 2.07 (1.42 to 3.01) |
| **Hemorrhagic stroke** |  |  |  |  |  |
| Pre-risk period | 4 | 20 | 3.57 (1.34 to 9.52) | 2.89 (1.87 to 4.49) | 0.61 (0.19 to 2.01) |
| Day 0 | 4 | 20 | 100.00 (37.53 to 266.44) | 2.89 (1.87 to 4.49) | 13.66 (3.90 to 47.83) |
| 1-90 days | 12 | 20 | 3.51 (1.99 to 6.17) | 2.89 (1.87 to 4.49) | 0.61 (0.15 to 2.45) |
| **Cerebral venous sinus thrombosis** |  |  |  |  |  |
| Pre-risk period | 1 | 5 | 2.10 (0.30 to 14.91) | 1.68 (0.70 to 4.05) | 1.60 (0.19 to 13.78)^c^ |
| Day 0 | 3 | 5 | 176.47 (56.92 to 547.16) | 1.68 (0.70 to 4.05) | 11.76 (26.41 to 472.92)^c^ |
| 1-90 days | 8 | 5 | 5.41 (2.71 to 10.82) | 1.68 (0.70 to 4.05) | 3.52 (1.15 to 10.76)^c^ |

CI denotes confidence interval, and COVID-19, coronavirus disease.

^a^Includes 3 additional individuals who received a COVID-19 vaccine between the infection and stroke dates, in addition to the 257 individuals excluded a priori for having a COVID-19 vaccination record within 49 days prior to stroke diagnosis.

^b^Incidence rate ratio estimated using the modified self-controlled case series method with a conditional Poisson regression model, adjusting for event-dependent censoring^17,18^ and calendar time.

^c^Incidence rate ratio estimated without adjustment for calendar time due to low case counts.

# **Table S3. Sensitivity analysis. Incidence of stroke following SARS-CoV-2 infection** **using either the pre-infection or post-infection period as the control window.**

|  | **Number of adverse events** | | **Event rate per 1,000 person-days** | | **Incidence rate ratio**  **(95% CI)**^a^ |
| --- | --- | --- | --- | --- | --- |
|  | **At risk** | **Control** | **At risk** | **Control** |  |
| **Considering only pre-infection control window** | | | | | |
| **Any stroke type** |  |  |  |  |  |
| Pre-risk period | 60 | 72 | 7.57 (5.88 to 9.75) | 2.84 (2.25 to 3.57) | 1.66 (1.10 to 2.50) |
| Day 0 | 32 | 72 | 113.07 (79.96 to 159.90) | 2.84 (2.25 to 3.57) | 24.31 (14.15 to 41.77) |
| 1-90 days | 119 | 72 | 4.98 (4.16 to 5.96) | 2.84 (2.25 to 3.57) | 1.28 (0.69 to 2.35) |
| **Pre-Omicron infection** |  |  |  |  |  |
| Pre-risk period | 26 | 29 | 6.07 (4.13 to 8.91) | 2.12 (1.47 to 3.05) | 2.03 (1.05 to 3.93) |
| Day 0 | 20 | 29 | 130.72 (84.33 to 202.62) | 2.12 (1.47 to 3.05) | 40.51 (19.31 to 85.01) |
| 1-90 days | 78 | 29 | 6.05 (4.85 to 7.56) | 2.12 (1.47 to 3.05) | 2.89 (1.18 to 7.10) |
| **Omicron infection** |  |  |  |  |  |
| Pre-risk period | 34 | 43 | 9.34 (6.67 to 13.07) | 3.68 (2.73 to 4.96) | 1.46 (0.86 to 2.49) |
| Day 0 | 12 | 43 | 92.31 (52.42 to 162.54) | 3.68 (2.73 to 4.96) | 12.78 (5.39 to 30.31) |
| 1-90 days | 41 | 43 | 3.73 (2.75 to 5.07) | 3.68 (2.73 to 4.96) | 0.52 (0.22 to 1.24) |
| **Ischemic stroke** |  |  |  |  |  |
| Pre-risk period | 55 | 59 | 8.25 (6.34 to 10.75) | 2.76 (2.14 to 3.57) | 1.87 (1.20 to 2.90) |
| Day 0 | 25 | 59 | 105.04 (70.98 to 155.45) | 2.76 (2.14 to 3.57) | 24.08 (13.22 to 43.86) |
| 1-90 days | 99 | 59 | 4.94 (4.05 to 6.01) | 2.76 (2.14 to 3.57) | 1.35 (0.70 to 2.60) |
| **Hemorrhagic stroke** |  |  |  |  |  |
| Pre-risk period | 4 | 11 | 4.61 (1.73 to 12.28) | 3.94 (2.18 to 7.12) | 0.69 (0.22 to 2.16)^b^ |
| Day 0 | 4 | 11 | 129.03 (48.43 to 343.79) | 3.94 (2.18 to 7.12) | 16.34 (5.20 to 51.33)^b^ |
| 1-90 days | 12 | 11 | 4.59 (2.61 to 8.09) | 3.94 (2.18 to 7.12) | 0.60 (0.26 to 1.38)^b^ |
| **Cerebral venous sinus thrombosis** |  |  |  |  |  |
| Pre-risk period | 1 | 2 | 2.55 (0.36 to 18.11) | 1.59 (0.40 to 6.35) | 0.95 (0.09 to 10.43)^b^ |
| Day 0 | 3 | 2 | 214.29 (69.11 to 664.41) | 1.59 (0.40 to 6.35) | 67.40 (11.26 to 403.35)^b^ |
| 1-90 days | 8 | 2 | 6.62 (3.31 to 13.23) | 1.59 (0.40 to 6.35) | 2.11 (0.45 to 9.97)^b^ |
| **Considering only post-infection control window** | | | | | |
| **Any stroke type** |  |  |  |  |  |
| Pre-risk period | 53 | 55 | 7.73 (5.90 to 10.11) | 2.56 (1.97 to 3.34) | 9.46 (3.04 to 29.40) |
| Day 0 | 29 | 55 | 118.37 (82.26 to 170.33) | 2.56 (1.97 to 3.34) | 127.22 (43.04 to 376.02) |
| 1-90 days | 108 | 55 | 4.90 (4.06 to 5.91) | 2.56 (1.97 to 3.34) | 5.60 (2.30 to 13.62) |
| **Pre-Omicron infection** |  |  |  |  |  |
| Pre-risk period | 23 | 25 | 6.08 (4.04 to 9.16) | 2.06 (1.39 to 3.05) | 6.15 (1.24 to 30.44) |
| Day 0 | 19 | 25 | 140.74 (89.77 to 220.65) | 2.06 (1.39 to 3.05) | 122.53 (26.32 to 570.46) |
| 1-90 days | 68 | 25 | 5.60 (4.41 to 7.10) | 2.06 (1.39 to 3.05) | 6.30 (1.82 to 21.83) |
| **Omicron infection** |  |  |  |  |  |
| Pre-risk period | 30 | 30 | 9.74 (6.81 to 13.93) | 3.22 (2.25 to 4.61) | 16.16 (2.93 to 89.16) |
| Day 0 | 10 | 30 | 90.91 (48.91 to 168.91) | 3.22 (2.25 to 4.61) | 115.49 (22.81 to 584.60) |
| 1-90 days | 40 | 30 | 4.04 (2.96 to 5.51) | 3.22 (2.25 to 4.61) | 4.48 (1.14 to 17.58) |
| **Ischemic stroke** |  |  |  |  |  |
| Pre-risk period | 49 | 42 | 8.62 (6.52 to 11.41) | 2.38 (1.76 to 3.22) | 8.65 (2.65 to 28.22) |
| Day 0 | 23 | 42 | 113.30 (75.29 to 170.50) | 2.38 (1.76 to 3.22) | 102.17 (32.53 to 320.87) |
| 1-90 days | 89 | 42 | 4.87 (3.96 to 6.00) | 2.38 (1.76 to 3.22) | 4.75 (1.89 to 11.96) |
| **Hemorrhagic stroke** |  |  |  |  |  |
| Pre-risk period | 3 | 10 | 3.97 (1.28 to 12.30) | 4.12 (2.21 to 7.65) | 2.33 (0.64 to 8.46)^b^ |
| Day 0 | 3 | 10 | 111.11 (35.84 to 344.51) | 4.12 (2.21 to 7.65) | 53.00 (14.59 to 192.57)^b^ |
| 1-90 days | 11 | 10 | 4.53 (2.51 to 8.17) | 4.12 (2.21 to 7.65) | 2.14 (0.91 to 5.05)^b^ |
| **Cerebral venous sinus thrombosis** |  |  |  |  |  |
| Pre-risk period | 1 | 3 | 2.38 (0.34 to 16.90) | 2.22 (0.72 to 6.89) | 2.59 (0.27 to 24.88)^b^ |
| Day 0 | 3 | 3 | 200.00 (64.50 to 620.11) | 2.22 (0.72 to 6.89) | 176.66 (35.66 to 875.25)^b^ |
| 1-90 days | 8 | 3 | 5.93 (2.96 to 11.85) | 2.22 (0.72 to 6.89) | 5.20 (1.38 to 19.58)^b^ |

CI denotes confidence interval, and COVID-19, coronavirus disease.

^a^Incidence rate ratio estimated using the modified self-controlled case series method with a conditional Poisson regression model, adjusting for event-dependent censoring^17,18^ and calendar time.

^b^Incidence rate ratio estimated without adjustment for calendar time due to low case counts.

# **References**

1 Altarawneh, H. N. *et al.* Effects of previous infection and vaccination on symptomatic Omicron infections. *The New England journal of medicine* **387**, 21-34, doi:10.1056/NEJMoa2203965 (2022).

2 Abu-Raddad, L. J. *et al.* Characterizing the Qatar advanced-phase SARS-CoV-2 epidemic. *Scientific reports* **11**, 6233, doi:10.1038/s41598-021-85428-7 (2021).

3 Chemaitelly, H. *et al.* Short- and longer-term all-cause mortality among SARS-CoV-2- infected individuals and the pull-forward phenomenon in Qatar: a national cohort study. *International journal of infectious diseases : IJID : official publication of the International Society for Infectious Diseases* **136**, 81-90, doi:10.1016/j.ijid.2023.09.005 (2023).

4 Altarawneh, H. N. *et al.* Protection against the Omicron variant from previous SARS-CoV-2 infection. *The New England journal of medicine* **386**, 1288-1290, doi:10.1056/NEJMc2200133 (2022).

5 Mahmoud, M. A. *et al.* SARS-CoV-2 infection and effects of age, sex, comorbidity, and vaccination among older individuals: A national cohort study. *Influenza and other respiratory viruses* **17**, e13224, doi:10.1111/irv.13224 (2023).

6 Abu-Raddad, L. J., Chemaitelly, H., Bertollini, R. & National Study Group for Covid Vaccination. Effectiveness of mRNA-1273 and BNT162b2 Vaccines in Qatar. *N Engl J Med* **386**, 799-800, doi:10.1056/NEJMc2117933 (2022).

7 Planning and Statistics Authority-State of Qatar. Qatar Monthly Statistics. Available from: <https://www.psa.gov.qa/en/pages/default.aspx>. Accessed on: May 26, 2020. (2020).

8 Chemaitelly, H., Bertollini, R., Abu-Raddad, L. J. & National Study Group for Covid Epidemiology. Efficacy of Natural Immunity against SARS-CoV-2 Reinfection with the Beta Variant. *N Engl J Med* **385**, 2585-2586, doi:10.1056/NEJMc2110300 (2021).

9 Chemaitelly, H. *et al.* Protection from previous natural infection compared with mRNA vaccination against SARS-CoV-2 infection and severe COVID-19 in Qatar: a retrospective cohort study. *The Lancet. Microbe* **3**, e944-e955, doi:10.1016/S2666-5247(22)00287-7 (2022).

10 Chemaitelly, H. *et al.* History of primary-series and booster vaccination and protection against Omicron reinfection. *Science advances* **9**, eadh0761, doi:10.1126/sciadv.adh0761 (2023).

11 AlNuaimi, A. A. *et al.* All-cause and COVID-19 mortality in Qatar during the COVID-19 pandemic. *BMJ Glob Health* **8**, doi:10.1136/bmjgh-2023-012291 (2023).

12 Akhtar, N. *et al.* Ischemic stroke in patients that recover from COVID-19: Comparisons to historical stroke prior to COVID-19 or stroke in patients with active COVID-19 infection. *PLoS One* **17**, e0270413, doi:10.1371/journal.pone.0270413 (2022).

13 Chemaitelly, H. *et al.* Association between COVID-19 vaccination and stroke: a nationwide case-control study in Qatar. *International journal of infectious diseases : IJID : official publication of the International Society for Infectious Diseases* **145**, 107095, doi:10.1016/j.ijid.2024.107095 (2024).

14 Akhtar, N. *et al.* Prolonged Stay of Stroke Patients in the Emergency Department May Lead to an Increased Risk of Complications, Poor Recovery, and Increased Mortality. *J Stroke Cerebrovasc Dis* **25**, 672-678, doi:10.1016/j.jstrokecerebrovasdis.2015.10.018 (2016).

15 Adams, H. P., Jr. *et al.* Classification of subtype of acute ischemic stroke. Definitions for use in a multicenter clinical trial. TOAST. Trial of Org 10172 in Acute Stroke Treatment. *Stroke* **24**, 35-41, doi:10.1161/01.str.24.1.35 (1993).

16 Bamford, J., Sandercock, P., Dennis, M., Burn, J. & Warlow, C. Classification and natural history of clinically identifiable subtypes of cerebral infarction. *Lancet* **337**, 1521-1526, doi:10.1016/0140-6736(91)93206-o (1991).

17 Farrington, C. P. *et al.* Self-Controlled Case Series Analysis With Event-Dependent Observation Periods. *J Am Stat Assoc* **106**, 417-426, doi:10.1198/jasa.2011.ap10108 (2011).

18 Fonseca-Rodríguez, O., Fors Connolly, A. M., Katsoularis, I., Lindmark, K. & Farrington, P. Avoiding bias in self-controlled case series studies of coronavirus disease 2019. *Stat Med* **40**, 6197-6208, doi:10.1002/sim.9179 (2021).
